# Effects of chewing gum against postoperative ileus after pancreaticoduodenectomy – a randomized controlled trial

**DOI:** 10.1186/s13104-015-0996-0

**Published:** 2015-02-10

**Authors:** Thomas Andersson, Kristofer Bjerså, Kristin Falk, Monika Fagevik Olsén

**Affiliations:** Department of Surgery, Sahlgrenska University Hospital, Gothenburg, S-41345 Sweden; Division of Nursing Science, Department of Medical and Health Sciences, University of Linköping, Linköping, Sweden; Institute of Health and Care Sciences, Sahlgrenska Academy at Gothenburg University, Gothenburg, Sweden; Department of Physical Therapy, Sahlgrenska University Hospital, Gothenburg, Sweden; Department of Gastrosurgical Research and Education, Sahlgrenska Academy at Gothenburg University, Gothenburg, Sweden

## Abstract

**Background:**

Postoperative ileus is common after surgery. One non-pharmacological intervention that has shown promising results in reducing the duration of postoperative ileus is chewing gum after surgery. However, this has not been investigated in upper gastrointestinal surgery such as pancreatic surgery. Hence the aim of this study was to investigate the effects of chewing gum treatment on patients undergoing pancreaticoduodenectomy ad modum whipple due to pancreatic or periampullary cancer.

**Methods:**

This study was conducted as a phase III trial that was terminated early. Patients diagnosed with pancreatic tumours scheduled for pancreaticoduodenectomy ad modum whipple were included. The treatment group received chewing gum postoperatively and standard care. Controls received glucose solution and standard care. Chewing gum and glucose were used four times a day during the whole hospital stay. Time to first flatus and stool was defined as the primary outcome. The secondary outcome was start with clear liquids, start with liquid diet and length of hospital stay.

**Results:**

No statistically significant differences could be observed between the chewing gum intervention group and the control group. However, a numerical difference in mean time was observed in first flatus, first stool, start of clear fluids, and start of liquid diet and length of hospital stay in favour of the intervention group.

**Conclusions:**

Although this study did not find statistically significant differences favouring the use of chewing gum for postoperative ileus, a positive trend was observed of a reduction of the impact of postoperative ileus among patients after pancreatic surgery. It also contributes valuable methodological experience that is important for future studies of chewing gum interventions during recovery after pancreatic surgery.

**Trial registration:**

ClinicalTrials.gov identifier: NCT02319512, publication date 2014-12-17.

## Background

Postoperative ileus (POI) is a common phenomenon after abdominal surgery. POI is the interval between surgery and passage of flatus/stool and tolerance of oral diet and prolonged POI is defined as “two or more of nausea/vomiting, inability to tolerate oral diet over 24 h, absence of flatus over 24 h, distention and radiologic confirmation on or after day 4 postoperatively without prior resolution” [1]. The main reason is considered to involve several factors such as: blocked extra cerebral signaling and the sympathic nervous system, inflammatory response (both local and systemic), and endocronological and hormonal effects [2]. Common symptoms of POI are vomiting, nausea and abdominal pain. Due to the high incidence of POI, it is considered a normal postoperative reaction [3]. However, prolonged POI results in an extended hospital stay and greater symptom burden for the patient [4].

Several strategies and interventions have been tested to prevent or reduce POI, both pharmacological and non-pharmacological. One of these strategies is gum chewing. Beneficial results have been shown in previous studies after urological, colorectal, gynecological and liver surgery [3,5]. These studies describe significantly reduced time to the first postoperative bowel movement [6-10], first flatus [6-11] and stool and shorter hospital stay after using chewing gum. Two meta-analyses [12,13] also describe beneficial effects on recovery after surgery. A possible mechanism for these results may be sham-feeding as a gastrointestinal response to neural and endocrine influence equivalent to that of eating but without the passage of food or fluid to the stomach.

Even though several studies have shown beneficial effects on recovery after different types of surgery, no study has yet investigated the effect of chewing gum treatment on recovery after major upper gastric surgery. Pancreaticoduodenectomy (PD) is considered a major surgical intervention with a high risk of prolonged POI and delayed gastric emptying (DGE). For this reason, the aim of this pilot study was to investigate the effects of chewing gum treatment on patients undergoing pancreaticoduodenectomy ad modum whipple due to pancreatic or periampullary cancer.

## Methods

This study was conducted as a phase III randomized, controlled, trial at a university hospital in Sweden. Patients diagnosed with pancreatic or periamullary cancer and scheduled to undergo pancreaticoduodencectomy ad modum Whipple with a panceraticogastrostomy and roux-en-y loop were included. Inclusion criteria were: understand and speak Swedish, no diagnosed neurological injuries or diseases affecting the ability to swallow or gastric function, no ongoing treatment for mental disease, no ongoing abuse of alcohol or other drugs, no previously known allergies to the contents of chewing gum. Primary outcomes were time to flatus/stool and secondary outcomes were length of hospital stay and start of clear liquids and fluid diet. Primary and secondary outcomes were assessed though patient records.

### Procedure

Participants were included on the day of admission, which was the day prior to surgery. Randomization to the two groups (1:1) was done when patients returned from the intensive care unit (ICU) to the ward. This procedure was chosen in order to avoid perioperative drop-outs due to inoperable tumors. A personnel from an independent institution not involved in the study administered pre-coded numbered, identical opaque envelopes to assign to the groups. A computer generated random table was used. The treatment group received chewing gum and standard care. Controls received standard care and sips of glucose, in total 3.6 g/day in a 12-ml mixture per day, the same amount of glucose per day as the treatment group received via the chewing gum. The chewing gum used was Chiza™, a natural organic gum whose main ingredients are latex, glucose and natural flavors (lime or spearmint). Patients started to chew after they returned from the ICU the day after surgery. Chewing gum was administered every fourth hour (08.00-12.00, 12.00-16.00 and 16.00-20.00). During each four-hour period, patients chewed two pieces of gum for 30 minutes. Chewing gum was used during the whole hospital stay. The time to the first postoperative flatulence and defecation was recorded, in addition to standard clinical data, by the first and the second authors.

Patients were monitored postoperatively at the ICU for 24 hours before returning to the surgical ward. All patients received a nasogastric tube perioperatively with cautious suction, which was kept for seven days. All patients received continuous thoracic epidural infusion of Bupivacain 1 mg/ml, Fentanyl 2 mg/ml and Adrenaline 2 mg/ml as an analgesic combined with intravenous paracetamol four times a day; all patients received a proton pump inhibitor (PPI) during the entire hospital stay and somastostatin for seven days postoperatively. When the nasogastric tube was removed, patients were allowed to start drinking a limited amount of clear liquids and later liquid foods (e.g. soups) and easily digested foods (e.g. fish). During fasting, patients received parenteral nutrition of glucose and Smofkabiven™ based on individual needs. Blood glucose levels were tested every fourth to sixth hour during the first postoperative week. When fluids could be tolerated, the epidural analgesia was converted to orally administered analgesia (Oxycodone and Paracetamol) until discharge based on the individual needs of the patient.

A standardized care plan focusing on rehabilitation, patient education, psychosocial needs and monitoring signs and symptoms was used in the postoperative nursing care to ensure equal and evidence based care.

### Statistics

Statistical power was calculated on the basis of results of the study by Add-El-Maeboud et al. [6] in which the mean value of time to the first flatulence was 17.9 h in the treatment group and 24.4 h in the control group. With a power of 80% and a level of significance of 0.05, 18 patients were needed in each group. As the variables were not normally distributed, comparisons between the groups were analyzed with non-parametric tests, the Mann Whitney *U* test and Chi 2 test. The statistically significant p value was set at <0.05.

Due to a radical change in postoperative care as well as surgical technique, adopting the ERAS protocol for pancreaticoduodenectomy [14], the study was terminated in advance as this change in care procedures differed extensively from the previous strategies, e.g. removal of the nasogastric tube much earlier in the postoperative phase and not using parenteral nutrition support as patients were allowed to start oral intake much earlier.

## Results

**Figure 1:**
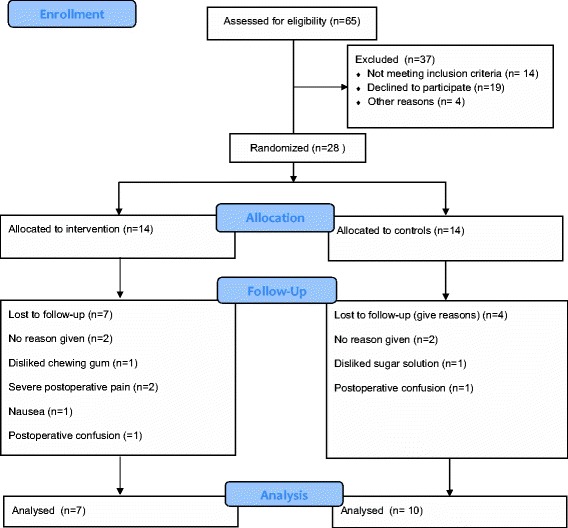
**Consort flowchart.**

Of 65 eligible patients, 51 fulfilled the inclusion criteria and were included in the study. Of the 65 eligible, 14 declined to participate for various reasons, e.g. not using chewing gum in their everyday life or difficulties coping with the postoperative situation in general and finding participation in a clinical study too much to deal with. A further 23 dropped out because of non resectable or additional surgery because of postoperative bleeding. The remaining 28 patients were randomized to the treatment group or the control group by opaque, sealed envelopes prepared by one person at the department who was independent of the study (Figure 1). Demographic data are presented in Table 1.

**Table 1:** **Demographic data in the intervention group and the control group**

	**Intervention n=7**	**Control n=10**	**Calculated difference (p)**
Gender (Male/Female)	5/2	4/6	.201
Age, years	65.9 (8.7)	63.2 (9.2)	.559
Duration of surgery, min	419.4 (96.1)	443.4 (97.4)	.0881
Peroperative blood loss, ml	971 (971.4)	1720 (1650.2)	.248

Number of participants or mean (±SD).

Of the total of 28 patients, 14 were randomized to the intervention group and 14 to the control group. Seven patients in the intervention group chose to discontinue participation in the study versus four in the control group (Figure 1). The majority of those discontinued participation because of postoperative symptoms such as pain, nausea or fatigue.

Of the remaining patients, there was a numerical difference in mean time to first flatus, first defecation, start of clear fluids, start of liquid diet and length of hospital stay in favour of the intervention group, although not strong enough to be statistically significant (Table 2).

**Table 2:** **Postoperative findings in the intervention group and the control group in mean (±SD)**

	**Intervention**	**Control**	**Calculated difference (p)**
Time to first flatus (SD) (days)	3.7 (1.4)	5.6 (4.4)	.340
Time to first defecation (SD) (days)	7.6 (2.7)	9.1 (6.2)	.882
Start of clear fluids (SD) (days)	5.1 (2.7)	7.7 (3.5)	.068
Start of liquid diet (SD) (days)	6.4 (2.7)	9.2 (3.6)	.116
Length of hospital stay (SD) (days)	18.0 (4.9)	21.8 (6.5)	.286

No side effects of chewing were recorded.

## Discussion

This is the first study of postoperative chewing gum to show a reduction of POI in major upper gastric surgery. Previous research has focused primarily on colon surgery with beneficial results on the duration of POI. Conducting a randomized controlled trial (RCT) with chewing gum in this patient group was associated with several difficulties, as demonstrated in the consort flow diagram (Figure 1). Fourteen potential participants declined participation. Reasons for being non participants were not registered; however almost 30% of the patients willing to participate were excluded because of advanced disease or a need of more extensive surgery, e.g. a total pancreatectomy, or were found to have advanced tumour growth, resulting in focusing on bypassing the tumour rather than removing it. Despite comprehensive preoperative screening, there is a risk of metastasis or vessel overgrowth not being detected by a CT scan, and some patients may therefore “slip” through the screening process only to be found to have an advanced disease that is detected perioperatively.

In this study, the mean time to first flatus and defecation was shorter in the intervention group, although the difference was not significant. This was also seen in the time to start intake of clear fluids and a liquid diet. While the hospital stay has been observed to be shorter, this study did not demonstrate any significant differences between the two groups. This may be related to the small number of participants and to the type of surgery and the impact on the patient’s ability to cope physically and mentally with its consequences for everyday life, not only to POI. Length of hospital stay was used as one outcome variable. However, in this type of surgery, there is a major risk of postoperative complications that may increase the length of the hospital stay.

A power analysis based on the results of the current study with 80% power and a significance level of 0.05 reveals that 37 patients per group is sufficient to show a significant change between groups concerning length of hospital stay, 46 for time to first flatus, 53 for start of clear fluids and liquid food and 160 patients per group for time to first defecation. Based on the results of this reverse power analysis, this study ought to be considered a preliminary report, and larger prospective trials are needed.

Patients randomized to the intervention group were affected by symptoms related to surgery such as pain, nausea or fatigue affecting their ability to tolerate the intervention. The use of a nasogastric tube with active suction for seven days was also demanding for the patients, further reducing their ability to cope. We also noted the importance of choosing the optimal chewing gum, considering texture, and of offering different flavours. Here, we used a biodynamic manufactured chewing gum containing glucose and not a sugar free alternative. This chewing gum had a somewhat different texture, and this might not appeal to everyone’s taste, thus resulting in drop-outs from the study. In the future it is important to focus on symptom management and to make the intervention as least intrusive as possible. While all the patients were very positive and interested in participating in our study, some were not able to continue participation because of their postoperative symptoms such as nausea, pain and fatigue.

## Conclusions

Even though this study does not demonstrate significant differences between the groups, it does indicate a positive trend of reducing the POI impact among patients after pancreatic surgery. It also contributes valuable experience for further studies on chewing gum interventions during recovery after pancreatic surgery.

### Ethics

This study was approved by The Regional Ethical Review Board of Gothenburg, Sweden, Dnr:886–11. The patients participated in the trial after written and verbal information was given and their written consent was obtained.

## Abbreviations

POI: Postoperative Ileus
PD: Pancreaticoduodenectomy
DGE: Delayed gastric emptying
RCT: Randomized controlled trial
ICU: Intensive care unit
PPI: Proton pump inhibitor
